# Quality of Nurses’ Communication with Mechanically Ventilated Patients in a Cardiac Surgery Intensive Care Unit

**DOI:** 10.17533/udea.iee.v37n2e02

**Published:** 2019-09-19

**Authors:** Marzieh Momennasab, Mohammadreza Shaker Ardakani, Fereshte Dehghan Rad, Roya Dokoohaki, Reza Dakhesh, Azita Jaberi

**Affiliations:** 1 Ph.D. Associate professor of nursing, Department of Nursing, Shiraz University of Medical Sciences, Shiraz, Iran. Email: momennasab@sums.ac.ir Shiraz University of Medical Sciences Shiraz Iran momennasab@sums.ac.ir; 2 M.Sc. Student, Shiraz University of Medical Sciences, Shiraz, Iran. email: azi635419@gmail.com Shiraz University of Medical Sciences Shiraz Iran azi635419@gmail.com; 3 . M.Sc. Nursing Instructor, Department of Nursing, Shiraz University of Medical Sciences, Shiraz, Iran. Email: dehghanrad@sums.ac.ir Shiraz University of Medical Sciences Shiraz Iran dehghanrad@sums.ac.ir; 4 M.Sc. Nursing Instructor, Department of Nursing, Shiraz University of Medical Sciences, Shiraz, Iran. Email:dokoohakir@sums.ac.ir Shiraz University of Medical Sciences Shiraz Iran dokoohakir@sums.ac.ir; 5 Nurse, B.Sc. Al-Zahra Hospital, Shiraz University of Medical Sciences, Shiraz, Iran. Shiraz University of Medical Sciences Shiraz Iran; 6 Ph.D. Assistant Professor of nursing, School of Nursing and Midwifery, Community Based Psychiatric Care Research Center, Shiraz University of Medical Sciences, Shiraz, Iran. Email: a_jaberi@sums.ac.ir. Corresponding author. Shiraz University of Medical Sciences Shiraz Iran a_jaberi@sums.ac.ir

**Keywords:** non-verbal communication, ventilators, mechanical, cardiac care facilities, patient satisfaction, intensive care units., comunicación no verbal, ventiladores mecánicos, instituciones cardiológicas, satisfacción del paciente, unidades de cuidados intensivos., comunicação não verbal, ventiladores mecánicos, institutos de cardiología, satisfação do paciente, unidades de terapia intensiva.

## Abstract

**Objective.:**

To describe the quality of the relationship between nurses and patients under mechanical ventilation.

**Methods.:**

This observational study, performed in a cardiac surgery intensive care unit in Iran, selected 10 nurses and 35 patients through simple random and convenience sampling, respectively. One of the researchers observed 175 communications between nurses and patients in different work shifts and recorded the results according to a checklist. Nurse and patient satisfaction with the communication was assessed by using a six-item Likert scale, 8 to 12 h after extubation.

**Results.:**

Most of the patients were male (77.1%), while most of the nurses were female (60%). Patients started over 75% of the communications observed. The content of the communication was related mostly to physical needs and pain. Besides, the majority of patients used purposeful stares and hand gestures, and head nod for communication. Most of the communications between patients and nurses were satisfied ′very low′ (45.7% in nurses, versus 54.3% in patients). However, ′complete satisfaction′ was lower in nurses (0%), compared with patients (5.7%). No statistically significant correlation was found between patients’ and nurses’ satisfaction and demographic variables.

**Conclusion.:**

The results showed that communication between nurses and mechanically ventilated patients was built through traditional methods and was based on the patients’ requests. This issue might be the cause of an undesirable level of their satisfaction with the communication, given that effective communication can lead to understanding and meeting the needs of the patients.

## Resumen

**Objetivo.:**

Describir la calidad de la relación entre las enfermeras y los pacientes sometidos a ventilación mecánica.

**Métodos.:**

Estudio observacional realizado en una Unidad de Cuidados Intensivos de Cirugía Cardíaca en Irán. Se seleccionaron 10 enfermeras y 35 pacientes mediante muestreo aleatorio simple y de conveniencia, respectivamente. Se observaron 175 comunicaciones entre las enfermeras y los pacientes en diferentes turnos de trabajo. Los resultados se registraron de acuerdo con una lista de verificación. La satisfacción con la comunicación de las enfermeras y de los pacientes se evaluó mediante una escala Likert de 6 ítems, de 8 a 12 horas después de la extubación del paciente.

**Resultados.:**

La mayoría de los pacientes eran hombres (77.1%), mientras que en las enfermeras predominó el sexo femenino (60%). El 75% de las comunicaciones observadas las iniciaron los pacientes. El contenido de la comunicación estaba relacionado principalmente con las necesidades físicas y el dolor. La mayoría de los pacientes usaron las miradas intencionadas, los gestos con las manos y los movimientos de la cabeza para comunicarse. La mayoría de las comunicaciones fueron ′ligeramente satisfactorias′ (45.7% para enfermeras versus 54.3% para pacientes). Sin embargo, la satisfacción general fue menor en las enfermeras (0%) en comparación con los pacientes (5.7%). No se encontró correlación estadísticamente significativa entre la satisfacción de los pacientes y de las enfermeras con las variables demográficas estudiadas.

**Conclusión.:**

Los resultados mostraron que la comunicación entre las enfermeras y los pacientes con ventilación mecánica se construyó a través de métodos tradicionales y se basó en las solicitudes de los pacientes. Esta situación puede ser el motivo del nivel indeseable de satisfacción con las comunicaciones, las cuales son esenciales para comprender y satisfacer las necesidades de los pacientes.

## Resumo

**Objetivo.:**

Descrever a qualidade da relação entre as enfermeiras e os pacientes submetidos a ventilação.

**Métodos.:**

Estudo observacional realizado em uma Unidade de Tratamento Intensivo de Cirurgia Cardíaca no Irã. Se selecionaram 10 enfermeiras e 35 pacientes mediante amostra aleatória simples e de conveniência, respectivamente. Se observaram 175 comunicações entre as enfermeiras e os pacientes em diferentes turnos de trabalho. Os resultados se registraram de acordo com uma lista de verificação. A satisfação com a comunicação das enfermeiras e dos pacientes se avaliou mediante uma escala Likert de 6 itens, de 8 a 12 horas depois da extubação do paciente.

**Resultados.:**

A maioria dos pacientes eram homens (77.1%), enquanto que nas enfermeiras predominou o sexo feminino (60%). 75% das comunicações observadas foram iniciadas pelos pacientes. O conteúdo da comunicação estava relacionado principalmente com as necessidades físicas e a dor. A maioria dos pacientes para comunicar-se usavam as olhadas intencionadas, os gestos com as mãos e os movimentos da cabeça. A maioria das comunicações foram ′ligeiramente satisfatórias′ (45.7% para enfermeiras versus 54.3% para pacientes). Porém, a satisfação completa foi menor nas enfermeiras (0%) em comparação com os pacientes (5.7%). Não se encontrou correlação estatisticamente significativa entre a satisfação dos pacientes e das enfermeiras com as variáveis demográficas.

**Conclusão.:**

Os resultados mostraram que a comunicação entre as enfermeiras e os pacientes com ventilação mecânica se construiu através de métodos tradicionais e se baseou nas solicitações dos pacientes. Esta situação pode ser o motivo do nível indesejável de satisfação com as comunicações, as quais são necessárias para compreender e satisfazer as necessidades dos pacientes.

## Introduction

Quality of Life (QoL) is defined as life conditions and satisfaction, which encompasses physical, psychological, social, and spiritual aspects. Interpersonal relationship is considered an important part of social aspects of QoL.(1) Effective communication is a basic part of qualified nursing care and is an important factor in playing an appropriate nursing role(2) in intensive care units (ICU), where the hospitalization experience is unpleasant.(3) Communication is an important factor to assess pain and other symptoms and for patients to participate in treatment decisions.(4) Some patients admitted to ICU are not able to speak and have difficulty communicating due to mechanical ventilation.(5) This problem can lead to anxiety, depression,(6) fatigue, frustration, hopelessness, and loss of control.(7) Some studies have also reported patient frustration and alienation due to failure in the communication process.(8,9) Evidence has also shown that in case patients are unable to express their symptoms, pain levels, and needs verbally, nurses will feel frustrated.(10)

Despite nurses’ high levels of knowledge and skills, they frequently confront problems in communicating with patients in ICU.(10) Additionally, many nurse-patient communications are task-oriented and aim at meeting patients’ immediate physical needs and performing clinical care.(11) Nevertheless, many studies have highlighted the importance and necessity of setting up communication between nurses and patients under mechanical ventilation. Researchers reported that critical care nurses devoted little time to communicating with their patients.(12) Difficulty in and failure of communication that reduces the amount of communication is mainly due to the inability to communicate verbally. When patients cannot respond to verbal communications, nurses do not often value talking with them, and interaction between nurses and patients will be based on the nurses’ ideas, assumptions, and previous notions about patients’ non-verbal behaviors.(10)

Ineffective communication can lead to patient and nurse dissatisfaction. The findings from a research on intubated patients’ satisfaction with methods of communicating with nurses showed that patients in a non-intervention group had low and high satisfaction levels.(13) Because nurses are among the most important part in communicating with patients,(14) they should pay attention to patients who try to communicate non-verbally.(15) Regarding the importance of the problems caused by communication failure and physical, mental, and spiritual needs of mechanically ventilated patients, this could have a negative impact on their recovery and might cause discomfort.(6) In studies conducted in Iran, barriers to the relationship between nurses and patients in cardiac surgery wards or mechanically intubated patients’ experiences have been investigated.(16,17) However, the quality of this relationship has not been particularly addressed in patients undergoing cardiac surgery and who were under mechanical ventilation. In other words, although the importance of communication between nurses and mechanically ventilated patients is obvious, little information is available regarding the quality of the current situation in ICU in Iran. Without this information, it will be difficult to define appropriate evidence-based standards for patients admitted to ICU without the ability to speak.(18) Achieving deeper knowledge from the quality of nurse-patient communication in ICU can help the nursing staff to gain a better understanding of the issue and provide more effective communication with these patients, given that communication can lead to the implementation of patient-based care, which, in turn, will reduce patient frustration.(19)

## Methods

This observational study was conducted in a cardiac surgery ICU with 14 beds in a cardio-specialized hospital in Shiraz, south of Iran from January to April 2014. This study is a part of a larger study, which sought to describe the quality of communication without communication aids between nurses and patients under mechanical ventilation in the ICU ward in Namazi hospital. In this research, 10 of the 21 qualified nurses were selected through simple random sampling (listing all nurses, assigning a number to each, and selecting numbers from a table of random numbers). The study inclusion criteria required nurses to have at least one year of working experience in ICU, be willing to participate in the research, and have the minimum required work shifts on a regular basis, which was four dayshifts and one nightshift. Additionally, the exclusion criteria included suffering from speech or hearing impairments, intending to quit the job, and moving to other hospital wards. According to a previous study,(20) 35 patients were required for this study. These patients were selected after cardiac surgery by using convenience sampling. The inclusion criteria for the patients included being between 18 and 60 years old, willing to participate in the study, having an endotracheal tube, being under mechanical ventilation, with Glasgow Coma Scale (GCS) score of 11 or above, and Richmond Agitation-Sedation Scale (RASS) score between -3 and +3. Furthermore, the exclusion criteria included decreased Glasgow score to below 11, RASS score > 3 or < -3, and suffering from blindness, deafness, and cognitive impairment.

The RASS was used to assess sedation and agitation(21) This scale can show changes in the level of consciousness over time or changes in response to analgesic drugs. The RASS scale is a 10-point numerical scale (+4 to -5). In this study, patients who obtained scores of +4, -4, and -5, respectively, indicating violence, deep sedation, and inability to respond to voice or physical stimulation, were excluded from analysis. The GCS is an assessment tool used to describe consciousness level;(22) it was developed to standardize observations of consciousness level in patients with head injuries. The scale consists of three separate responses: eye opening (E), verbal (V), and motor responses (M); each classified by a series of degrees of responsiveness. Each subdivision was allocated a number, and higher numbers mean better scores. The data collected contained demographic characteristics (age, sex, marital status, education level, and ability to read and write), data of admission, reason for surgery, language, hearing, and vision status, substance abuse, mental disorders, recent surgery, GCS score, and RASS score. 

This study also assessed communication quality, including content of communications (five contents), communication methods (four methods), and the initiator of the communications (nurse or patient) by using a researcher-prepared observation checklist. The checklist was developed based on the existing literature in psychological and behavioral domains(20,23) and was approved by faculties of Nursing. The content of communication included physical needs, pain, symptoms, emotions, decisions on treatment, and questions about the endotracheal tube. In addition, communication methods included head nod, non-verbal expressive actions, and writing. Besides, the initiator of the communication was either the nurse or the patient. The checklist was filled during and after the communication. Moreover, nurse and patient satisfaction with communication during routine care in ICU were evaluated by a six-item Likert scale, ranging from “completely satisfied” to “not at all satisfied”. Five faculties of Nursing experienced in critical care nursing approved the validity of the scales. However, inter-rater reliability of the observation checklist has not been evaluated. At first, the nurses selected became familiar with the study procedure, signed written informed consents to take part in the study, and completed demographic information forms in one session. Then, the patients waiting for cardiac surgery were selected based on the study’s inclusion and exclusion criteria using convenience sampling. After explaining the study objectives and procedures and obtaining written informed consents to participate in the study, demographic information forms were completed by the patients. After cardiac surgery, GCS and RASS scales were completed for the patients and those qualified were enrolled in the study.

One of the researchers, who was in the staff, observed routine communications between the nurses and patients in different work shifts and recorded the results according to the checklist. The presence of the observer could affect nurses’ behavior and their relationship with patients. However, a researcher attending several meetings before formal observations might turn him to *the participant as observer* according to Gold's Typology of Participant-Observer Roles.(24) Overall, the study recruited 35 patients and 10 nurses. Five communications from each nurse with patients were observed; therefore, the observer monitored 175 patient-nurse communications. Patient satisfaction was assessed 12 h after extubation. Nurse satisfaction was also evaluated after each communication.

The Ethics Committee of the Shiraz University of Medical Science approved this study. Moreover, all participants signed written informed consents after receiving an oral explanation about the research objectives and procedures. Participants were also assured about anonymity and confidentiality of their information. The data obtained was analyzed by using the SPSS software, version 22. Descriptive statistics, including mean, frequency, and standard deviation, were applied to describe demographic, professional, and communication features. Additionally, the correlations among the variables were assessed by using T-test, one-way ANOVA, chi-square test. To determine the correlation between the patients’ satisfaction level and the demographic parameters, given that the data were not distributed normally, Mann-Whitney U and Wilcoxon non-parametric tests were used.

## Results

The majority of the patients were male (77.1%) and all were married. Patients’ ages ranged from 24 to 60 years, with mean age of 52.05 years. Furthermore, most of the patients (82.8%) had undergone coronary artery bypass graft surgery (Table 1). In addition, 60% of the nurses were female and their ages ranged from 26 to 49 years, with mean age of 31±6.64 years. In addition, the mean of their working experience was 3.55±0.98 years at ICU and 7.94±5.82 years as a nurse. Eight nurses (80%) held BSc. degrees and two (20%) had MSc. degrees.

**Table 1:** General Characteristics of patients (n=35)

Characteristics	Values
Age in years; mean ±SD, range	52.05±8.56, 24 - 60
Males; n (%)	27 (77.1)
Marital status; n (%)	
Married	35 (100)
Education level; n (%)	
Uneducated	10 (28.6)
Undergraduate and Graduate	25 (65.7)
Academic	2 (5.7)
Substance abuse; n (%)	
No	26 (74.3)
Yes	9 (25.7)
Surgery; n (%)	
Coronary Artery Bypass Graft	29 (82.8)
Valve surgery	6 (17.2)

Patients initiated 75.43% of the communications observed (132/175), whereas nurses only did it in 24.54% (43/175), mostly to check consciousness level and encourage patients to breathe. Considering communication contents, 50.3% was related to physical needs - eating, drinking, elimination, oral care, and positioning, 23.5% was related to pain, and only 1.1% was related to the patients’ feelings, including frustration, anxiety, and fear (Table 2). Moreover, most of the communication methods used by patients (88.57%) were non-verbal expressive actions, including hand gestures and purposeful stares, followed by head nods (10.86%), with only one instance of writing by the nurse (0.57%). Assistive communication tools, such as word and picture boards, were not used.

**Table 2:** Content of 175 communications between nurses and intubated patients

Content	Percent
Physical needs	50.3%
Pain	23.5%
Emotions	1.1%
Endotracheal Tube Therapy questions	17.2%
Other symptoms	1.1%

Considering participant satisfaction, the results showed that only 5.7% of the patients and 0% of the nurses were “completely satisfied” with the communications. However, the nurses showed less overall satisfaction, compared to the patients. According to the results, 20% of the nurses showed no satisfaction (Table 3). 

**Table 3:** Frequency distribution of the nurse and patient satisfaction levels with the communications

Level	Nurses (n = 10)	Patients (n = 35)
Completely	0%	5.7%
Partially	2.8%	8.6%
Low	31.4%	22.8%
Very low	45.7%	54.3%
Not at all	20.0%	8.6%

Table 4 shows no significant relationships between the nurses’ satisfaction and their working experience as nurses in ICU, and patients’ age, gender, education level, and history of substance abuse and similar finding in the patients’ satisfaction level with the demographic parameters.

**Table 4:** Relationship between the satisfaction of nurses and patients with demographic and professional characteristics by group

		Group	
Characteristics	Nurses		Patients
	p-value		p-value
Nurses			
Age	0.983		0.250
Gender	0.582		0.727
Education	0.598		0.769
Experience	0.885		0.782
Patients			
Age	0.943		0.785
Gender	0.873		0.277
Education	0.630		0.087
Substance abuse	0.697		0.076

## Discussion

The findings of this study disclosed that patients initiated communication in more than three fourths of the cases, whereas nurses initiated communication when checking the patients’ consciousness and encouraging them to breathe. These findings contrast those from other studies wherein nurses initiated communication with intubated patients.(20) These differences could be attributed to different working conditions and to the fact that the nurses in those studies used communication aids. Due to challenges in communicating with and understanding patients, nurses in the present study might have avoided contact with them. Results from other studies also indicate that nurses became hopeless and, consequently, avoided contact when patient-nurse communication was difficult.(16) Other factors, including heavy workload,(25) lack of appropriate communication skills training, and lack of communication aids are also among the effective factors in this regard.(26)

In our study, basic physical needs, pain, and discomfort were the main reasons driving intubated patients to establish communication. These findings are similar to those from another study.(2) The findings of the current study reveal that only a small percentage of the content of the nurses’ communication with intubated patients (1.14%) involved emotions and feelings. The findings also indicate that the majority of the communications involved the patients’ physical rather than emotional needs. In this sense, other studies have disclosed that, although important, the patients’ emotional needs were treated subsequent to their physical needs.(27) Yet, it is clear that if patients’ physical needs are satisfied, they will express their emotional conditions, such as anxiety, discomfort, and frustration.(28) When nurses initiate communication and there are more instances of communications, patients’ emotional and spiritual needs, *i.e*., requirements of holistic care, are taken into account besides their physical needs.(29) Failure to pay attention to emotional and mental aspects not only prolongs length of hospital stay, but also aggravates the disease status and leads to discomfort, anxiety, and frustration.(30)

To maintain communication, most patients in the present study used non-verbal expressive actions, such as purposeful stares, hand gestures, and head nods. This was consistent with findings from other studies wherein most patients used body language and purposeful stares.(20) According to studies, non-verbal behaviors, such as squeezing a hand,(2) head nod, and gesture,(31) were the most common methods used by ICU nurses and patients to communicate (2). Although these methods can be accompanied by misinterpretation,(32) some studies have shown that proper use of these techniques can facilitate communication. During the current study, researchers observed only one instance of using pen and paper in response to patient’s request. The nurses apparently relied on their experience to comprehend these patients’ messages. Nevertheless, having access to communication aids and training the staff on the use of said aids could significantly increase their communication success. To communicate with their patients, nurses can also use augmentative and alternative communication strategies.(15,31)

Regarding satisfaction, 20% of the nurses were “not at all satisfied” with the communication. However, the rest of the nurses reported partial satisfaction to very low satisfaction with communication. Nurses’ dissatisfaction could be due to failure in understanding the patients’ needs and inefficient communication. Thus, nurses’ satisfaction levels can be increased by increasing their knowledge and skills in communicating with intubated patients and providing them with access to communication aids, which lead to communication that is more successful. On the other hand, patients showed lower satisfaction levels. This could be because the nurses’ failure in comprehending the patients’ messages entailed non-fulfillment of their needs. This agreed with results from other studies, indicating that the majority of the patients (62.2%) were poorly satisfied with their communications, while the patients’ satisfaction levels significantly increased by using different methods to facilitate communication.(13) Nurses and patients’ low satisfaction levels, and poor usage of communication aids indicated the need to pay more attention to communication with intubated patients, nurses’ training, and provide required communication aids in Iran.

### Limitation.

One of the limitations of the present study was the presence of the observer, which might have affected nurses’ behaviors. Of course, given that the observer’s role was to participate as observer, which is common in health care settings, observing operations could help to understand and improve care processes, including communications. It should also be noted that inter-rater reliability of the observation checklist has not been evaluated. Therefore, considering this issue should be warranted in future studies. Moreover, because this study took place in only one ward, it is hard to generalize the results. Thus, further studies are recommended on larger sample sizes at different centers.

## Conclusion.

The results of the present study indicate that nurses’ communication with mechanically ventilated patients in cardiac surgery ICU was run in a traditional way (non-verbal with no communication aids), mostly focused on physical needs and not emotional needs, and it was not satisfactory. Teaching these issues and communication aids to Iranian nurses and nursing students is poorly described in most Iranian studies and requires longer follow-up studies to implement comprehensive care based on the needs of patients in intensive care unit, both in prevention and in nursing interventions. 
